# Increasing temperatures accentuate negative fitness consequences of a marine parasite

**DOI:** 10.1038/s41598-020-74948-3

**Published:** 2020-10-28

**Authors:** Sean C. Godwin, Mark D. Fast, Anna Kuparinen, Kate E. Medcalf, Jeffrey A. Hutchings

**Affiliations:** 1 Department of Biology, Dalhousie University, 1355 Oxford Street, Halifax, NS B3H 4R2 Canada; 2 Atlantic Veterinary College, University of Prince Edward Island, Charlottetown, PE Canada; 3 Department of Biological and Environmental Sciences, University of Jyväskylä, Jyväskylä, Finland; 4 Institute of Marine Research, Flødevigen Marine Research Station, His, Norway; 5 Centre for Coastal Research, University of Agder, Kristiansand, Norway

**Keywords:** Ecology, Climate-change ecology, Ecological epidemiology

## Abstract

Infectious diseases are key drivers of wildlife populations and agriculture production, but whether and how climate change will influence disease impacts remains controversial. One of the critical knowledge gaps that prevents resolution of this controversy is a lack of high-quality experimental data, especially in marine systems of significant ecological and economic consequence. Here, we performed a manipulative experiment in which we tested the temperature-dependent effects on Atlantic salmon (*Salmo salar*) of sea lice (*Lepeophtheirus salmonis*)—a parasite that can depress the productivity of wild-salmon populations and the profits of the salmon-farming industry. We explored sea-louse impacts on their hosts across a range of temperatures (10, 13, 16, 19, and 22 °C) and infestation levels (zero, ‘low’ (mean abundance ± SE = 1.6 ± 0.1 lice per fish), and ‘high’ infestation (6.8 ± 0.4 lice per fish)). We found that the effects of sea lice on the growth rate, condition, and survival of juvenile Atlantic salmon all worsen with increasing temperature. Our results provide a rare empirical example of how climate change may influence the impacts of marine disease in a key social-ecological system. These findings underscore the importance of considering climate-driven changes to disease impacts in wildlife conservation and agriculture.

## Introduction

Infectious disease can be a major driver of population declines in wildlife^1,2^ and mortality events in agriculture^3,4^. From the collapse of bat populations in eastern North America due to white-nose syndrome^5^ to the tremendous economic damages experienced by the Indian livestock industry due to brucellosis^6^, it is becoming ever more apparent that infectious disease plays a key role in determining wildlife population dynamics and agriculture production. Climate change is expected to shift the geographic distributions of many pathogens and parasites of wildlife^7^ and livestock^8^, but whether climate change will cause an overall increase in diseases globally has been a topic of major controversy (reviewed by Rohr et al. (2011)). One of the main limitations in assessing the potential responses of disease to climate change has been a lack of high-quality data from experiments with adequate replication, temperature treatments, and hierarchical analyses^9,10^. Over the past decade and a half, further experimental work has clarified that climate change may have severe consequences for disease progression^11,12^ and transmission^13,14^, pathogen growth^15,16^, and host susceptibility^17,18^, but less is known about how climate might influence the *impacts* (i.e., fitness consequences) of disease on hosts.

Climate change has a disproportionate influence on oceans and coastal communities^19,20^, but the effects of climate on the ecology of infectious marine diseases is generally not well understood^21^. Despite recent catastrophic declines in corals (e.g., *Montastraea* spp.^22^), sea stars (e.g., *Pisaster ochraceus*^23^), abalones (e.g., *Haliotis rufescens*^24^), and other marine ectotherms^25^—all associated with disease and high ocean temperatures—there has been little experimental work testing how marine disease and temperature interact to impact hosts^21^. A handful of experiments have observed temperature-dependent effects of parasites on host survival^13,26,27^, a couple of which elegantly integrated the survival results with thermal performance curves or transmission data to develop a more holistic understanding of host-parasite dynamics under future environmental change^28,29^. However, rarely have such investigations coupled survival data with other measures of fitness, especially in systems of key ecological and economic importance. This lack of experimental focus on interactions between temperature and marine-disease impacts is surprising; marine diseases are remarkably influential, spreading much more rapidly than their terrestrial counterparts^30^ and shaping the structure and function of ecosystems^31,32^. Marine diseases in commercially important species cause massive financial losses each year, particularly in the aquaculture sector where high densities of stressed hosts can provide ideal conditions for disease to spread^33^. Whether and how climate change will affect the impacts of disease on fisheries and aquaculture is a critical question given fishery-catch plateaus in recent decades and the world’s increasing reliance on seafood protein^34^, but one that remains unanswered.

Farmed and wild salmon (*Salmo salar* and *Oncorhynchus* spp.) comprise an important social-ecological system that exemplifies the complexities of marine-disease dynamics and the need to understand the effects of climate change on disease impacts. Marine open-net salmon farming is the most lucrative form of aquaculture globally^35^, but over the past two decades the economic and ecological sustainability of the industry has been challenged by a group of ectoparasites called sea lice (primarily *Lepeophtheirus salmonis*). Every year, the salmon-farming industry loses approximately 9% of revenues due to sea lice—in Norway alone equivalent to $436 million USD in damages—mainly through treatments for controlling louse outbreaks and decreased salmon biomass^36^. Sea lice can reduce the survival^37,38^, growth rate^39,40^ and body condition^41^ of their hosts. Consequently, sea-louse transmission between farmed and wild salmon can also negatively impact wild-salmon populations^42–44^, many of which have immense cultural, economic, and ecological value. Unlike salinity^45,46^, temperature doesn’t have a strong effect on louse survival in the temperature ranges observed naturally. Sea lice do, however, have temperature-dependent development rates^47,48^, so outbreaks in wild and farmed salmon increase in frequency and severity in warmer waters^49–51^, which may further reduce host survival^52^. However, it is entirely unknown whether the effects of sea lice on their hosts will also change as ocean temperatures rise, despite the obvious consequences for the salmon-farming industry and wild-salmon populations.

Here, we provide a rare experimental example of how climate change can affect the impacts of marine disease in an important social-ecological system. We performed a manipulative experiment across three infestation levels (i.e., zero, ‘low’, and ‘high’) and five temperatures (10, 13, 16, 19, and 22 °C) to test whether water temperature influences the effects of parasitic sea lice on the growth, condition, and survival of juvenile Atlantic salmon. We predicted that the effects of the parasites on their hosts would be exacerbated by increasing temperature.

## Results

The mean louse abundance at the end of the experiment was 1.4 lice per fish (95% CI: 1.2, 1.7) for the low-infestation treatment and 6.8 lice per fish (6.2, 7.4) for the high-infestation treatment. Only adult lice and a small number of pre-adult 2 lice were present at the end of the experiment (Fig. S1). There was no evidence of reproduction (i.e., copepodite or chalimus-staged lice), as expected given the flow-through nature of the tanks. Neither louse abundance nor louse survival had an obvious relationship with temperature for either infestation level, with a couple notable exceptions; for high-infestation fish at the midpoint dissections, the lowest midpoint louse abundance was found in the 10 °C treatment, while low-infestation fish at 22 °C and high-infestation fish at 19 °C had the highest endpoint louse abundance and midpoint-to-endpoint louse survival in their respective infestation levels (Fig S4).

For each of our three response variables—growth rate (measured as g degree-day^−1^), change in condition, and survival—the effect of infestation on the response worsened with increasing temperature and infestation level. The best-supported models for growth rate, change in condition, and  survival all included fixed effects for infestation level, temperature, and an interaction between the two.

**Figure 1:**
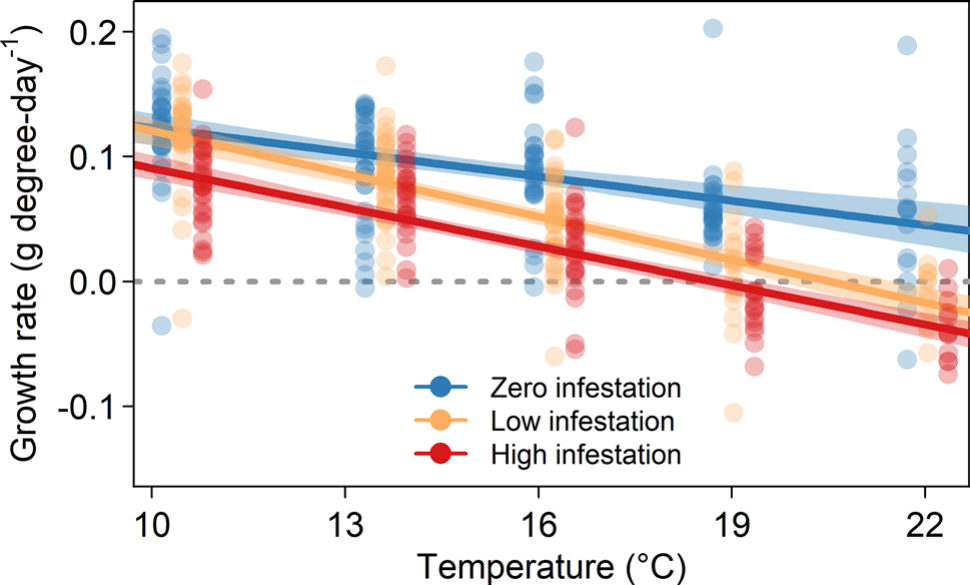
Growth rates of Atlantic salmon post-smolts as a function of infestation level and temperature. The points depict observed growth rates (jittered by infestation level for visualization purposes) and the lines describe the mean predictions from the top linear mixed-effects model (with 95% bootstrapped confidence intervals).

The growth-rate model that included an interaction between infestation level and temperature had overwhelming statistical support, as it was 9.89 AIC units lower than the second-ranked model and had an AIC weight of 0.99 (Table S1). Growth rate decreased linearly with temperature for all infestation levels (Fig. 1). Fish in the low-infestation level experienced the strongest effect of temperature on growth rate (Table S4). There was no difference in predicted growth rate between the zero- and low-infestation levels at lower temperatures, but by 12.7 °C the difference was statistically clear (0.105 g degree-day^−1^ [95% CI: 0.097, 0.114] for zero infestation and 0.090 g degree-day^−1^ [0.082, 0.097] for low infestation). Fish in the high-infestation level experienced consistently depressed growth rates relative to zero-infestation fish, in addition to a stronger temperature effect than in the zero-infestation level (Table S4; Fig. 1). The mean predicted growth rates were negative in temperatures warmer than 20.6 °C for low-infestation fish and 18.7 °C for high-infestation fish.

**Figure 2:**
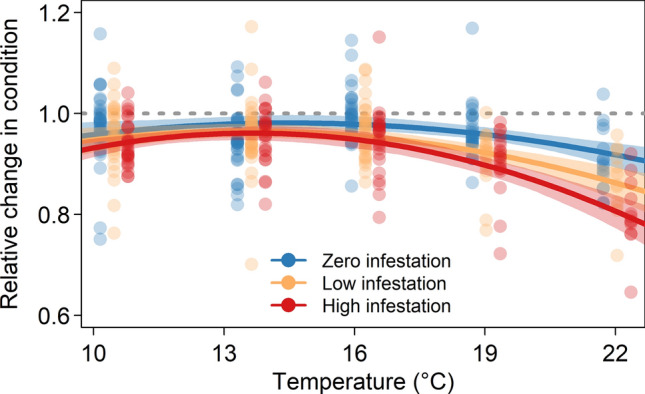
Condition of Atlantic salmon post-smolts at the end of the experiment relative to the start. The points represent observed change-in-condition values (jittered by infestation level for visualization purposes) and the lines give the mean predictions from the top change-in-condition model (with bootstrapped 95% confidence intervals) across the range of experimental temperatures for each of the three infestation levels.

The top change-in-condition model, for which temperature was modelled as a quadratic term, was 3.13 AIC units lower than the second-ranked model and accounted for 83% of model support (Table S2). Predicted change in condition (i.e., condition at the end of the experiment relative to the start) was statistically indistinguishable among infestation levels at temperatures colder than 16.7 °C for low-infestation fish and 15.8 °C for high-infestation fish. In warmer temperatures, however, low-infestation and high-infestation fish had reduced change-in-condition values relative to zero-infestation fish (Fig. 2). This interaction between infestation and temperature was particularly evident at the high-infestation level; by 22 °C, predicted change in condition was on average 14% lower for high-infestation fish than zero-infestation fish (0.92 [95% CI: 0.89, 0.94] for zero infestation and 0.81 [0.77, 0.84] for high infestation).

**Figure 3:**
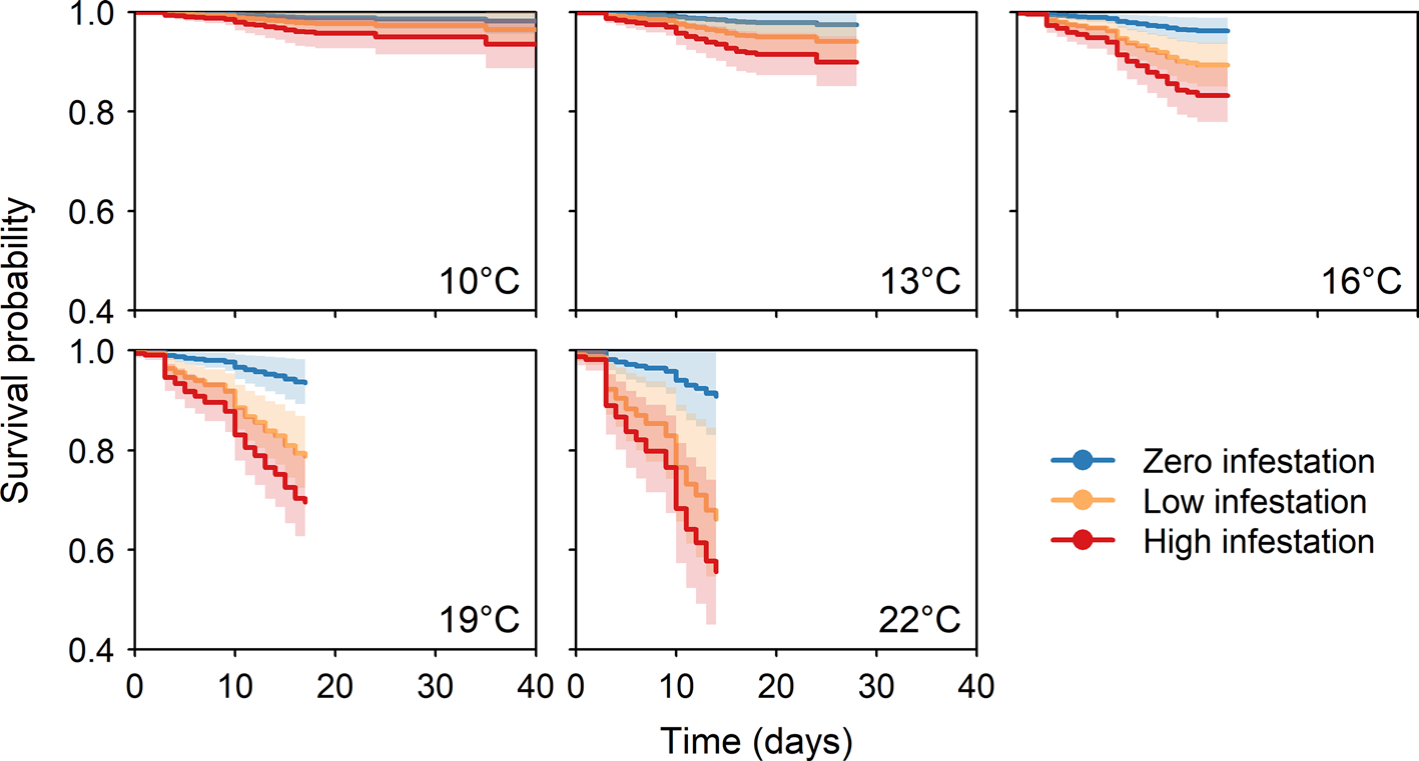
Survival curves for Atlantic salmon post-smolts across three infestation levels and five temperatures. In total, there were 82 mortalities during the experiment. The curves are constrained to the duration of the experiment, which differed among temperature treatments because sea-louse development rate increases with temperature. Since the statistical framework for generating survival curves from mixed-effects Cox models has yet to be developed, we show the survival curves from a basic Cox model with the same fixed effects as our top model.

The top survival model indicated that temperature and infestation level decreased survival probability of Atlantic salmon and that the two effects interacted to decrease survival further (Fig. 3). This model was 3.70 AICc units lower than the second-ranked model and had 86% of model support (Table S3). In total, 82 of the original 768 fish died during the experiment: 9 zero-infestation fish, 29 low-infestation fish, and 44 high-infestation fish (Table S4). Hazard ratios (i.e., the multiplicative increase in chance of mortality relative to zero-infestation fish at 10.5 °C) increased with temperature for all infestation levels, but this temperature effect was stronger for low-infestation fish than for zero-infestation fish, and stronger still for high-infestation fish (Fig. S2). At 16 °C, the mean predicted hazard ratios for zero-, low-, and high-infestation fish were 3.0, 4.1, and 8.7, respectively, whereas at 22 °C they were 9.8, 20.3, and 37.3.

## Discussion

Our results provide experimental evidence of how climate change can alter the impacts of marine disease. We found that the consequences of sea-louse infestation for captive juvenile Atlantic salmon are mediated by temperature. As temperatures increased, so too did the effects of sea lice on the growth rate, condition, and survival of their hosts.

The temperatures and infestation levels used in our experiment were realistic given expected increases in coastal ocean temperatures and observed louse abundances on salmon farms. In Atlantic Canada, where we conducted our experiment, mean near-surface water temperatures typically sit between 10 and 17 °C in the summer, while maximum near-surface water temperatures already often exceed 22 °C (i.e. our highest experimental temperature)^53^. By the middle of this century, mean sea-surface temperatures in this region are projected to increase roughly 4 °C relative to temperatures at the turn of the twenty-first century (except in Labrador where projections indicate a 2 °C increase)^54^, indicating that our highest experimental temperatures were empirically meaningful, given current and projected temperatures. Similarly, our infestation levels were well within the range of observed sea-louse abundances on salmon farms in Atlantic Canada; in fact, the mean adult-female abundance from our high-infestation treatments (6.8 ± 0.4 lice per fish) was actually lower than the majority of adult-female counts on salmon farms in Atlantic Canada in recent years^55,56^. It is difficult to compare our experimental sea-louse abundances to those experienced by wild juvenile salmon in Atlantic Canada due to a lack of publicly available data, but the abundances we observed fall well within the range found on wild Atlantic salmon in Norway, another Northern-Hemisphere country with a large salmon-farming industry^57^.

The mechanisms that led to the observed temperature-dependent effects of sea lice are somewhat unclear. While temperature accelerates louse development^47,48^ and can therefore increase louse abundance^49,58^ and the frequency and severity of epizootics^50^, survival of attached lice doesn't have a strong relationship with temperature as it does with salinity^45,46^, at least in natural temperature ranges. In theory, temperature-dependent differences in host resistance (i.e., their ability to reduce their parasite burden^59^) or host tolerance (i.e., their ability to ameliorate the damage caused by infestation^59^) could lead to the results we observed—if, at higher temperatures, more sea lice survive to adulthood or lice cause more damage to their hosts, we would expect the effects of sea lice to worsen with increasing temperature. However, the evidence from our experiment for either of these mechanisms was equivocal. While the lowest midpoint louse abundance for high-infestation fish was at 10 °C and the highest endpoint louse abundance and survival was at 19 °C for low-infestation fish and at 22 °C for high-infestation fish, these patterns weren’t consistent across temperatures (Fig. S3), suggesting that temperature-dependent resistance may have played a role but was probably not the main driver of our results. It is not clear whether temperature-dependent tolerance had a strong influence on the impacts of sea lice as we have no measures of tolerance at the physiological level and because our only measure of ‘damage’ caused by lice had the opposite relationship with temperature than we would have expected; louse-induced skin lesions on surviving fish at the end of the experiment were least severe at the highest temperatures (Fig. S4), although this could well have been a product of the shorter duration of infestation or higher mortality from lesions at higher temperatures. Still, it would be intuitive that at higher temperatures salmon would have less energy to allocate to immune response due to lower feed conversion efficiency and higher metabolic demands^60^. Our results provide a foundation for further immunological research to clarify whether and how host resistance and tolerance influence temperature-dependent impacts of parasite infestation.

Two events disrupted the intended course of the experiment and could conceivably have influenced our results. First, a logistical issue delayed our infestation date after we had already adjusted temperatures to their assigned levels, forcing us to correct the temperatures back to the ambient level for fourteen days to minimize the effects of temperature-dependent growth prior to infestation. While it is possible that this initial temperature exposure before the experiment had a minor, exacerbating effect on the subsequent host-parasite interactions in the high-temperature treatments, we think this is unlikely given the two-week re-acclimatization period and the fact that pre-exposure to stressful events tends to prime fish and other animals to subsequent exposure to those stressors, especially in the case of temperature^61–64^. If the pre-exposure to high temperatures did, in fact, prime our fish for the experimental temperatures, our results could be seen as conservative. The second event was Hurricane Dorian, which caused temperature fluctuations immediately after infestation in the three temperature treatments that were manually rather than electronically adjusted (Fig. S5). Temperature variability can alter disease transmission and impacts^65,66^, so these fluctuations, while consistent within temperature treatments (Fig. S5), may have influenced our results. The temperature treatments affected were the three coldest ones, so any negative effect of increased temperature variance on growth, condition, or survival should have diminished the observed temperature-dependent impacts of sea lice rather than accentuated them.

The economics of the salmon-farming industry stand to be affected by an increase in sea-louse impacts to domesticated Atlantic salmon. Decreased growth, condition, and survival of farmed fish would likely result in reduced profits^36^. Adapting to climate-driven increases in the effects of sea lice may be an underappreciated challenge for the industry in upcoming years, largely because existing solutions are limited. These include increasing the duration of stocking to offset growth reductions, harvesting fish at smaller sizes (thus decreasing their market value), or increasing the frequency of delousing treatments^39^. The latter option is unlikely because the industry is already having to contend with widespread resistance of sea lice to parasiticides^67^, so increasing the frequency of treatments would not only be expensive, but also potentially accelerate the development of resistance. Emerging solutions for reducing louse infestations (e.g., new sea-cage technologies and widespread use of farmed cleaner fish) may play an important role in the future of parasite management on farms, but their disadvantages will have to be addressed before they can realistically offset climate-driven increases to louse impacts (e.g., infrastructure costs for new cage technologies and welfare issues for cleaner fish).

There could also be substantial ecological consequences of climate-driven increases in the effects of sea lice on their hosts. In Europe, large-scale experiments have suggested that sea-louse infestation reduces recruitment for wild-salmon populations^43,44,68^, so an increase to louse-induced direct and indirect mortality (via reductions to early marine growth and condition^69,70^) could have profound impacts at the population level. While acknowledging that the fish in our experiment were of hatchery origin and therefore any inference about the applicability of our results to wild fish has that caveat, it is also possible that these effects could compound with other effects of climate on wild salmon (e.g., habitat deterioration^71^ and the uncoupling of predator–prey phenology^72^). From a conservation perspective, climate-driven impacts may be particularly important for small, already-imperilled populations that are more sensitive to environmental stochasticity^73^—a concern given that most Atlantic salmon populations and many Pacific salmon populations are but fractions of their historical abundances^74–77^. How exactly our results might be applied to on-the-ground conservation efforts is not immediately obvious. Rising ocean temperatures are already expected to increase the frequency and severity of louse outbreaks^50^ and decrease host survival due to faster louse development^52^, and our results suggest that climate warming will likely have an even greater effect on this host-parasite system than previously expected. While further work would be needed to extrapolate this experimental work to wild populations, it is conceivable that in order to maintain the same level of impact on wild salmon that farms might have to manage sea lice to lower levels in the future, at least in warm months or years.

Marine diseases can devastate populations of wild and farmed aquatic organisms, but they can be challenging to study because disease detection, mitigation, manipulation, and monitoring are all generally more difficult in the ocean than in terrestrial environments. Accordingly, research on interactions between marine disease and climate is often performed as an emergency response to an outbreak or a post-mortem to a disease-induced population crash^78^. It makes sense, therefore, that most of these studies are observational, with the intent of deciphering which climate correlates best predict disease responses^10,21^. Observational studies have the benefit of placing disease relationships in their ecological context, but unlike experimental work they are unable to identify causal links or separate the effects of climate on disease development, prevalence, and severity. The lack of experimental work on this topic has been one of the greatest challenges to our understanding of how climate influences marine disease^9,21^.

The question of how climate change influences infectious diseases is now well into its second decade of controversy. Despite the growing body of experimental work showing the influence of temperature on disease development and transmission in wildlife^11–16^, investigation into how climate change might influence disease *impacts* on hosts remains surprisingly limited (but see^26–29^), especially for ecologically and economically important marine systems. Our results help fill this key knowledge gap by providing strong evidence for climate-driven increases to marine-disease impacts in an important social-ecological system, as well as a foundation for predictive modelling work to build upon^79^. As it becomes ever more obvious that infectious disease is a key driver of wildlife populations and agriculture production, understanding how climate governs disease impacts may be vital for effective conservation and resilient agricultural systems in this era of rapid environmental change.

## Methods

### Experiment

To assess the temperature-dependent effects of sea lice on juvenile Atlantic salmon, we experimentally manipulated sea-louse infestation on Atlantic salmon post-smolts across three infestation levels (i.e., zero, ‘low’, and ‘high’) and five temperatures (10, 13, 16, 19, and 22 °C). Each combination of infestation level and temperature was triplicated, resulting in a total of 45 tanks. The fish were 23.6 ± 0.1 cm (mean ± SE) in fork length and 143.7 ± 1.2 g in weight at the beginning of the experiment (Fig. S7). We performed our experiment at the Aquatron facility of Dalhousie University in Halifax, Canada.

We sourced Saint John River strain Atlantic salmon post-smolts from Cape d’Or Sustainable Aquaculture, at whose facilities the fish were reared for 18 months from eyed ova in typical groundwater-fed and temperature-controlled hatchery conditions. At the time of sourcing, this particular strain had been raised in captivity at commercial sites for approximately 17 generations. We initially held the fish in eight 1750 L recirculating seawater tanks (radius = 92 cm, height of water line = 66 cm) at 12 °C. After allowing the fish 12 days to acclimatize, we anaesthetized them in a 100 mg L^−1^ tricaine methanesulfonate (MS-222) seawater solution and inserted PIT tags (7 × 1.35 mm; FDX-B, Loligo Systems) into their abdominal cavities via a small (2–3 mm) intraperitoneal incision^80^. Ten days after surgery, we divided the fish into 45 × 70 L flow-through seawater tanks (radius = 21 cm, height of water line = 46 cm), each with an adjustable air bubbler at the bottom. We randomized this relocation by first moving the salmon in batches into a 300 L fish tote (length = 97 cm, width = 55 cm, height = 58 cm) by dipnets and then transferring fish from the tote into the experimental tanks individually and sequentially. We held the tanks at ambient seawater temperature (~ 13 °C) and randomly assigned each tank to their future temperature treatment (one of 10, 13, 16, 19, and 22 °C).

Twenty-five days after transferring the fish to the experimental tanks, we began adjusting the water temperatures by a maximum rate of 1.5 °C day^−1^ until each tank reached its assigned temperature. After allowing ten days of acclimatization at the new temperatures, we adjusted the tank temperatures back to the ambient level due to delays in louse collection—again at a maximum rate of 1.5 °C day^−1^—to minimize the influence of temperature-dependent growth on the size and condition of the fish before infestation. Fourteen days later, the tanks were adjusted back to their assigned temperatures at the same rate of change. After ten days of re-acclimatization at the new temperatures, we anesthetized the fish in the same manner as for the PIT tag surgeries and then weighed them and measured their fork lengths. We allowed three days for recovery from the anesthetic before infesting the fish with sea lice.

We infested the fish by exposing them to copepodid (i.e., larval) *L. salmonis* as described in Poley et al.^81^ and Whyte et al.^82^. Briefly, adult female lice with egg strings were collected from sea-cage sites in Bay of Fundy, New Brunswick (NB), in August 2019. Egg strings were removed and cultured in flow-through systems at the Huntsman Marine Science Centre (St. Andrews, NB, Canada). After nine days in culture, we received a stock seawater mixture with a known concentration of copepodites from Huntsman and divided this mixture among the tanks according to their randomly assigned infestation level. For the low-infestation tanks, we drew from the stock mixture a volume equivalent to 15 lice per fish, topped it up to 150 mL, and poured it into the tank. For the high-infestation tanks, we drew from the stock mixture a volume equivalent to 70 lice per fish. Tanks at the zero-infestation level were given 150 mL of plain seawater. Once a tank was infested, we left it for 70 min to allow lice to attach before turning the flow back on. We infested 15 tanks at a time (one from each combination of temperature and infestation level), with 20 min between each set of infestations. At the time of infestation, the number of fish in each tank ranged from 12 to 20 (mean ± SE = 18 ± 2 fish), depending on random variation in background mortality over the holding period, for a total of 768 fish.

Each tank was equipped with a temperature logger (HOBO Pendant MX2201, Onset) that we submerged 5 cm from the surface and recorded measurements every three minutes. We fed the fish 3 mm pellets (Nutra RC, Skretting) at a rate of 1% body weight per day and monitored the tanks daily for mortalities, air input, and flow rates (mean ± SE = 1.40 ± 0.14 L min^−1^). We opportunistically measured salinity (range: 28–32 ppt) and pH (range: 7.2–7.7) in the tanks and measured dissolved oxygen saturation every 1–2 days (89.5 ± 0.3%).

Within one day of infesting the salmon, Hurricane Dorian made landfall in Halifax, Canada. The storm resulted in heavy fluctuations in the ambient seawater temperature, unexpected equipment issues, and on-the-ground logistical problems for the technicians responsible for maintaining our experimental temperatures. Consequently, our lower three temperature treatments—those that were manually adjusted rather than electronically—were more variable and, on average, warmer than anticipated for the first ten days of the experiment (Fig. S5). We still observed clear separation in temperature among the assigned temperature treatments and over the course of the experiment the mean observed temperature for each treatment (10.5, 13.6, 16.3, 19.0, and 22.0 °C) was within 0.6 degrees of the assigned temperature (10, 13, 16, 19, and 22 °C).

Once the lice developed into pre-adults, the timing of which varied depending on temperature, we euthanized and dissected approximately 40% of the fish in each tank (hereafter called ‘midpoint dissections’; n = 286). The number of fish removed for the midpoint dissections varied among tanks depending on initial numbers and mortalities. We euthanized the remaining fish after the lice developed into adults (hereafter called ‘endpoint dissections’; n = 400). Since louse development rate is governed by temperature^48,83^, the timing of the endpoint dissections varied according to temperature treatment (from 14 days for the 10 °C treatment to 40 days for the 22 °C treatment; Fig. S5). Fish of all infestation levels within a temperature treatment were euthanized on the same day. We determined this timing using the temperature-dependent development data for sea lice from Hamre et al. (2019), who report the mean number of degree-days between infestation and adulthood for male and female lice across seven temperatures between 3 °C and 21 °C. To calculate the number of degree-days to adult for *our* temperatures, we linearly interpolated and extrapolated the Hamre et al. data (averaged between the sexes) and added an arbitrary 30% additional time to ensure that all lice had molted to adults. We euthanized the fish with a 250 mg L^−1^ overdose of MS-222, after which we weighed them, measured their fork lengths, and froze them at − 20 °C. We assessed the sea-louse infestation of the thawed fish under a dissecting microscope by counting each louse and identifying its sex and life stage. Louse abundances reported here include both male and female lice (but see Fig. S1 for abundances grouped by sex and life stage).

All work conducted with animals for this project was approved by the Dalhousie University Committee on Laboratory Animals under protocol #19-015, in accordance with Canadian Council on Animal Care guidelines.

### Statistical analysis

We calculated fish growth rates during the experiment as wet weight per degree-day. Using degree-days rather than calendar days allowed us to compare growth rates among temperature treatments, which would have otherwise been impossible given the decrease in experiment length at higher temperatures due to faster louse development. Since fish growth generally scales linearly with cumulative degree days^84,85^, as was the case specifically for the fish in our experiment (Fig. S6), this choice of growth metric minimizes the confounding effect of temperature on the growth results. We calculated the cumulative number of degree-days (*CDD*) for each temperature treatment in the standard manner:

1 $$CDD = \mathop \sum \limits_{d = 0}^{n} \left( {\frac{{T_{{max_{d} }} + T_{{min_{d} }} }}{2} - T_{0} } \right)$$

where *d* is the number of days since infestation (*d* = 0), *n* is the duration of the experiment in days, $T_{{max_{d} }}$ and $T_{{min_{d} }}$ are the maximum and minimum temperatures for a given day, and $T_{0}$ is the base temperature (i.e., the temperature below which growth is effectively zero^84^). We used a $T_{0}$ value of 0 for three reasons: (1) it is a recommended standard^84^, (2) there is published precedent for assuming $T_{0} = 0$ for juvenile Atlantic salmon^86^, and (3) precise $T_{0}$ estimates are generally unnecessary^84^. We calculated growth rates (*GR*) as

2 $$GR = \frac{{W_{end} - W_{start} }}{CDD}$$

where *W*_*start*_ and *W*_*end*_ are the wet weights of the fish in grams at the start and end of the experiment and *CDD* is the cumulative number of degree-days (from Eq. 1) that the fish experienced.

We fitted five linear mixed-effects models to these growth-rate data (Table S1), including only the fish that survived to the end of the experiment and excluding 27 fish that lacked initial body weight data (n = 373). The most complex model included fixed effects for infestation level, temperature, and the interaction between the two. The remaining models included the other four combinations of these parameters (including a null model with none of them) because all of these combinations were determined a priori to be biologically plausible. We treated temperature as a continuous covariate, using the mean observed temperatures from the experiment. The models all had a variance structure that allowed for unequal variance in growth among infestation levels, as was observed. Every model included a random effect on the intercept for tank to account for the hierarchical structure of the experimental design. We performed model selection, using the Akaike Information Criterion (AIC;^87^).

We calculated the relative condition factor (K; hereafter called ‘condition’) for each fish at the beginning and end of the experiment as described by Le Cren^88^:

3 $$K = \frac{W}{{aL^{b} }}$$

where *W* is the wet weight of the fish in grams, *L* is the fork length of the fish in millimetres, *a* is the exponentiated intercept of the log–log length–weight relationship of the experimental fish at the end of the experiment, and *b* is the slope of the same length–weight relationship (Fig. S7). The relative change in condition ($\rho$) over the course of the experiment was therefore

4 $$\rho = \frac{{K_{end} }}{{K_{start} }}$$

where *K*_*start*_ and *K*_*end*_ are the conditions of the fish at the start and end of the experiment.

We fitted the same five linear mixed-effects model forms to these change-in-condition data as we did for the growth rates, with the exception that no variance structure was included because variance was relatively constant among infestation levels. We fit these models to the data from fish that survived to the end of the experiment (n = 373), excluding only those for which we lacked initial body weights (n = 27). We also fitted three additional models that included quadratic terms for temperature (Table S2). We conducted model selection on these eight models, using the AIC.

We fitted five mixed-effects Cox proportional hazards models to the survival data from the experiment (n = 82), using and the same fixed- and random-effects structure as for the growth models (Table S3). We treated fish that were removed in the midpoint or endpoint dissections as censored data. We performed model selection using AIC corrected for small sample sizes (AICc;^89^).

We repeated the growth-rate, change-in-condition, and survival analyses using the average temperature in each tank as a correlate rather than the average temperature in each temperature treatment; the results were unchanged (Tables S7–S9). We bootstrapped confidence intervals for the responses by sampling each tank with replacement, using the original sample size, re-fitting the top model to this sample dataset, calculating the mean predictions for each infestation level across the range of temperatures, repeating this process 10,000 times, and calculating the 2.5th and 97.5th percentile of the 10,000 mean predictions. All analyses were conducted in R version 3.6.1^90^ using the nlme^91^ and coxme^92^ packages.

## Supplementary information

Supplementary Information

## Data Availability

The full dataset for this study can be downloaded from the Dryad Digital Repository at 10.5061/dryad.2jm63xskg.
