# Supplementary material for: Increasing temperatures accentuate negative fitness consequences of a marine parasite
Source: Sci Rep. 2020 Oct 28;10:18467. doi: 10.1038/s41598-020-74948-3 (PMC7595087; doi:10.1038/s41598-020-74948-3)
Supplement: Supplementary file 1 — Supplementary Information [file 41598_2020_74948_MOESM1_ESM.docx]

**Supplementary Information**

**Title:** Increasing temperatures accentuate negative fitness consequences of a marine parasite

**Authors:** Sean C. Godwin, Mark D. Fast, Anna Kuparinen, Kate E. Medcalf, Jeffrey A. Hutchings


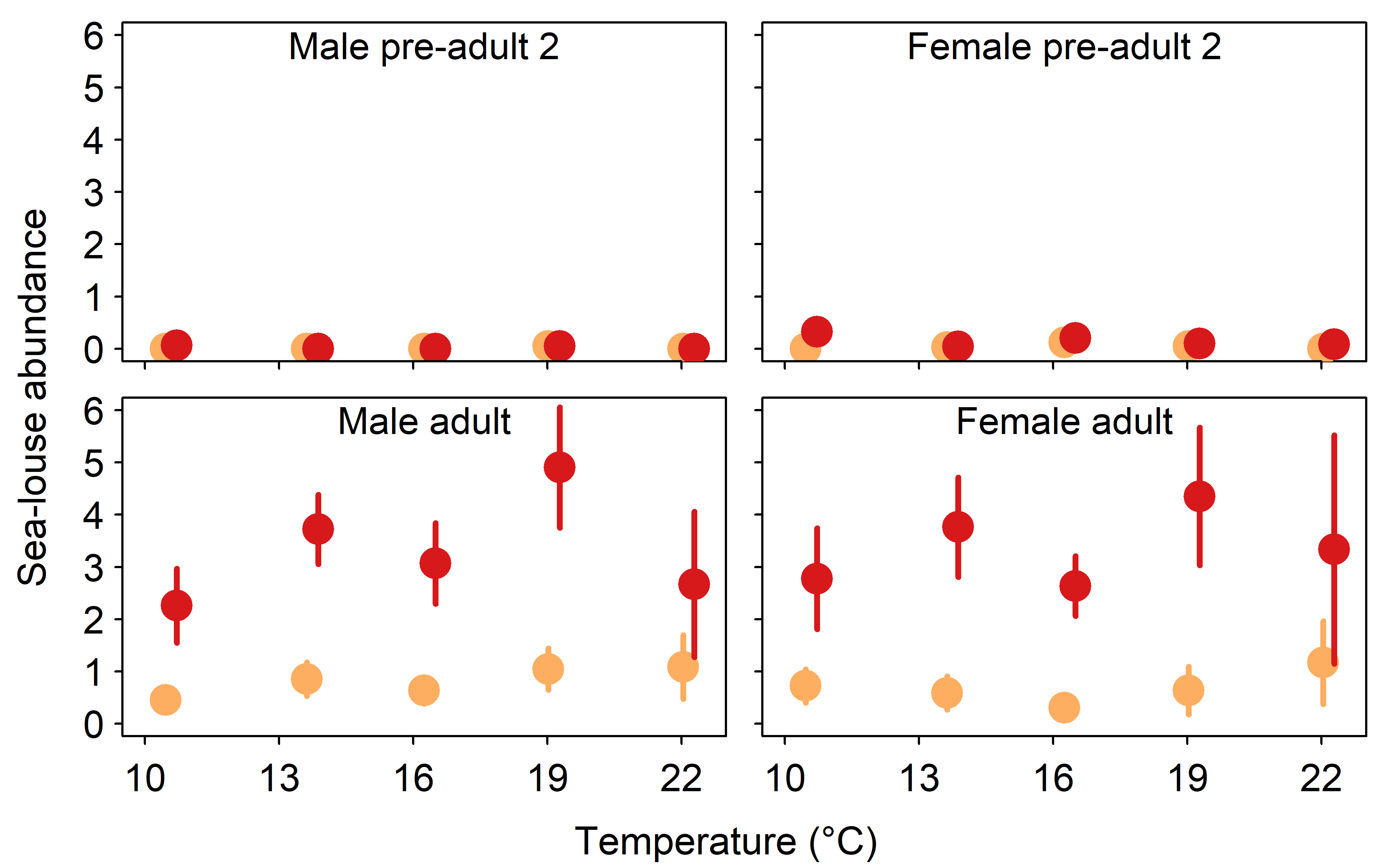
Fig. S1. Abundance (with bootstrapped 95% confidence intervals) of sea-louse life stages and sexes at the end of the experiment for each temperature treatment and infestation level.


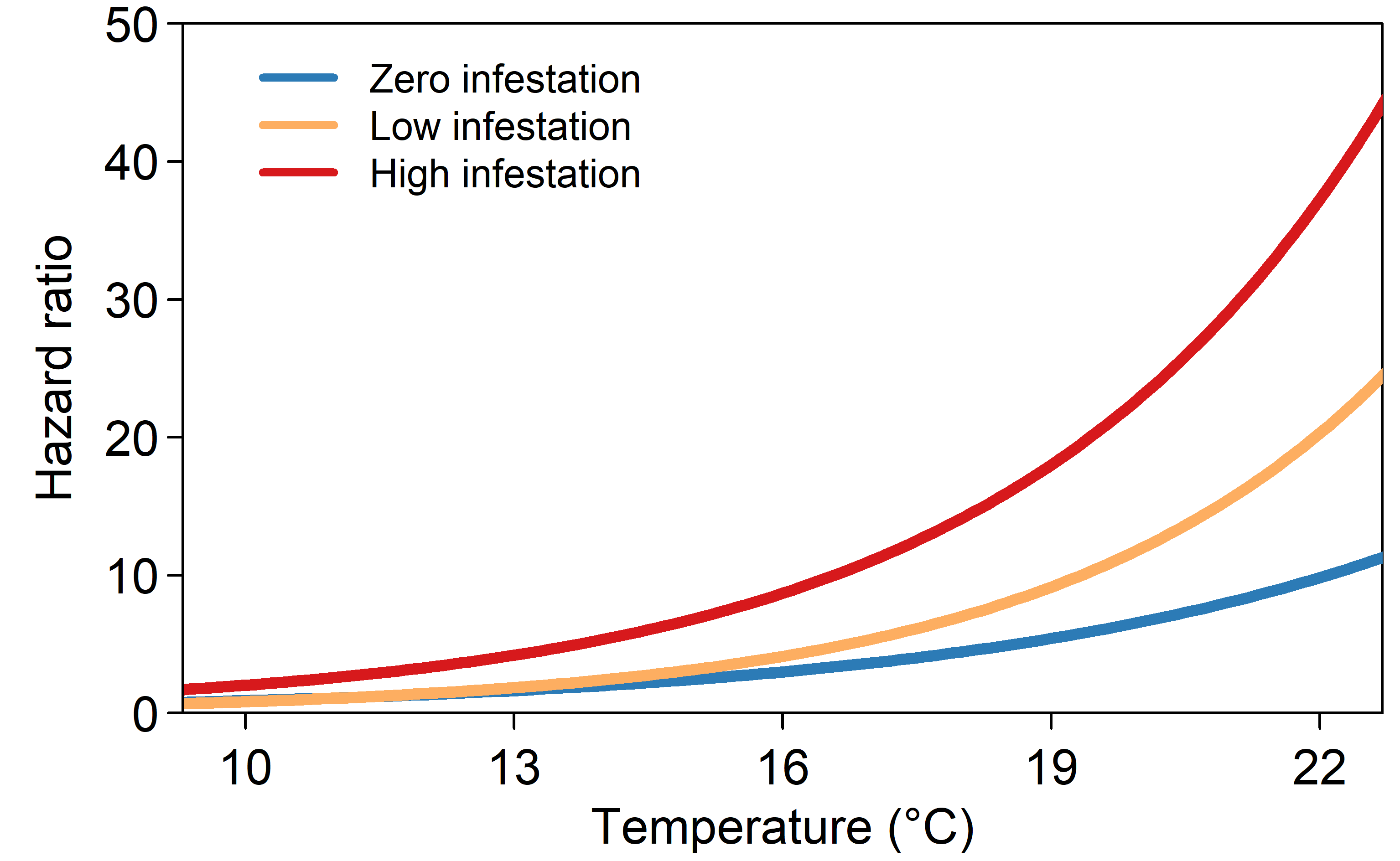


Fig. S2. Hazard ratios across the range of experimental temperatures for each of the three infestation levels. These hazard ratios can be interpreted as the multiplicative increase in the chance of mortality relative to zero-infestation fish at 10.5 °C.


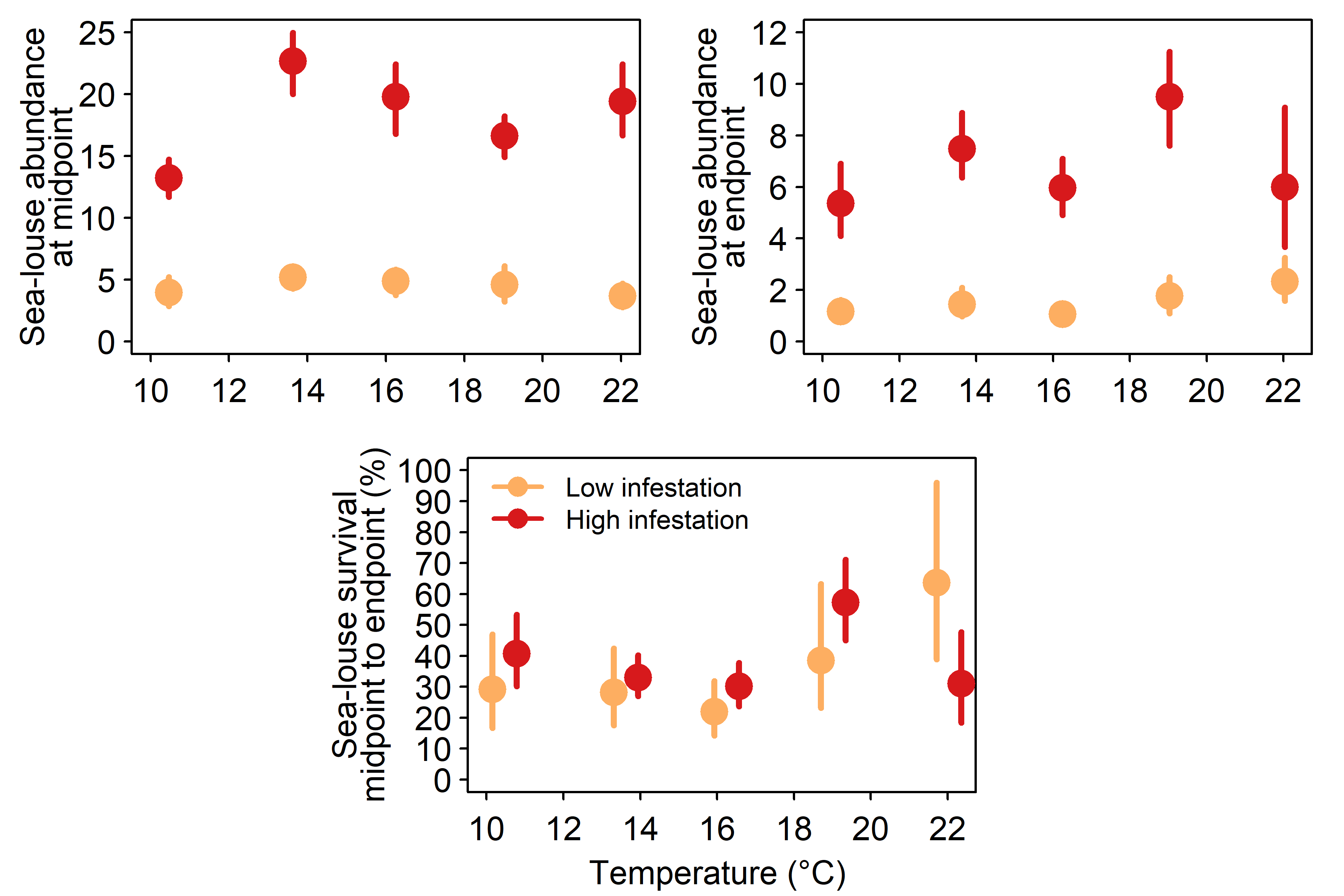


Fig. S3. Median sea-louse abundance at the midpoint dissections (top-left panel), sea-louse abundance at the endpoint dissections (top-right panel), and sea-louse survival from midpoint to endpoint dissections (bottom panel) for each temperature and infestation level. Data are jittered for visualization purposes only. Error bars give the bootstrapped 95% confidence intervals.


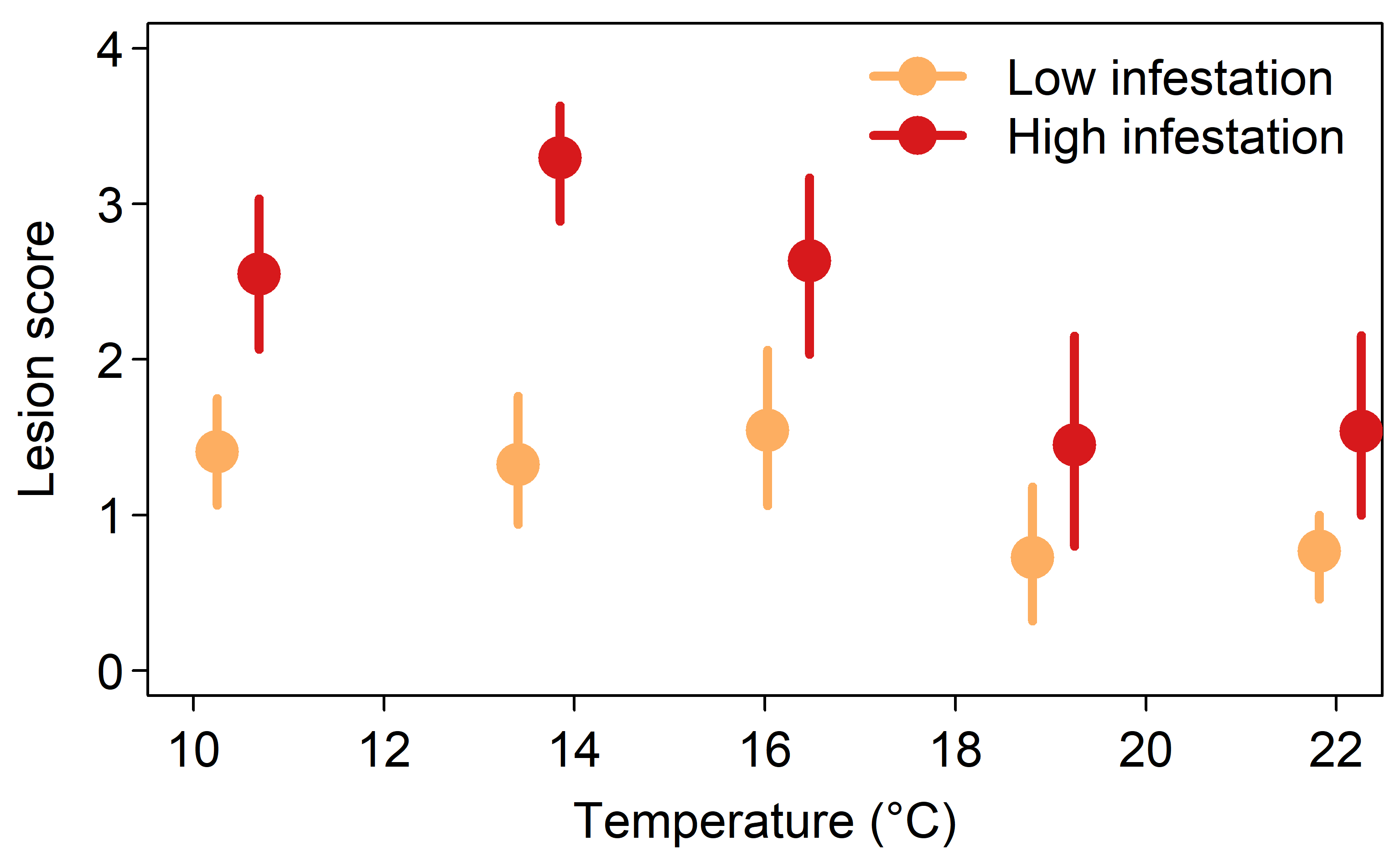


Fig. S4. Median skin-lesion scores (with bootstrapped 95% confidence intervals) across the experimental temperatures and infestation levels. Lesion development and severity was assessed through gross analysis of skin on the head and area posterior to the dorsal fin using a scoring system modified from Johnson et al. (1996). Skin was scored based on a scale from 0 to 6, where 0 = normal skin, 1 = skin discolouration ≤ 1 cm diameter, 2 = skin discolouration > 1 cm diameter, 3 = erosion and scale loss ≤ 1 cm diameter, 4 = erosion and scale loss > 1 cm, 5 = lesion reaching the musculature or deeper ≤ 1 cm diameter, and 6 = lesion reaching the musculature or deeper > 1 cm diameter. Data are jittered for visualization purposes only.


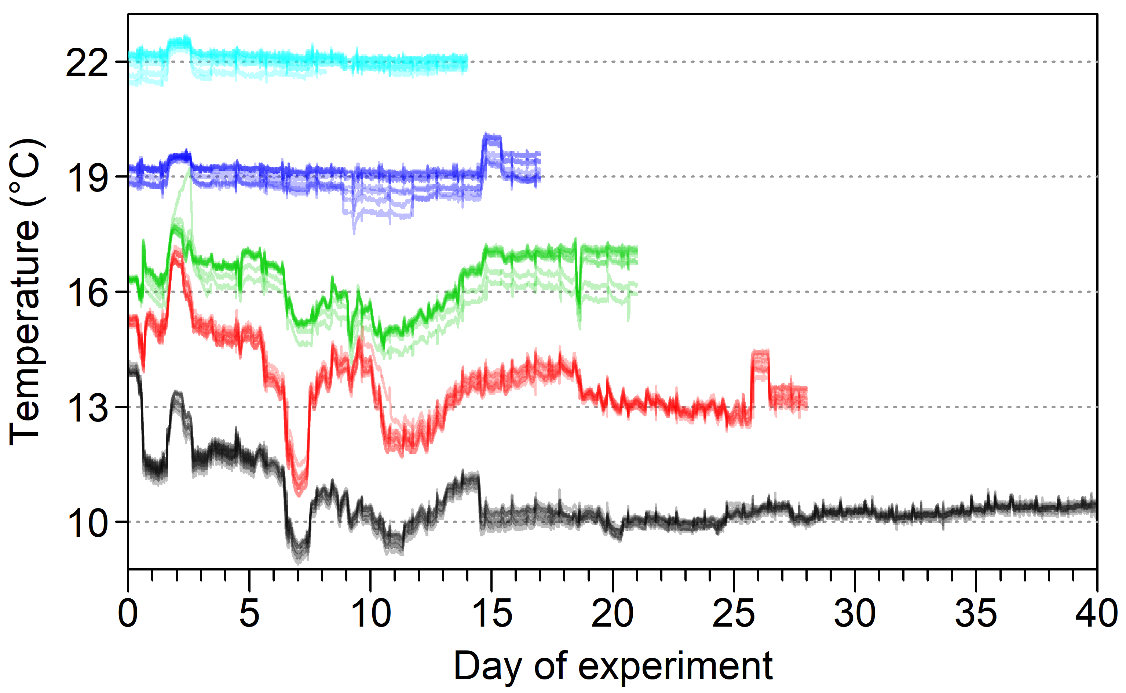


Fig. S5. Experimental temperatures from infestation (day = 0) to endpoint dissections, which were performed when sea lice reached adulthood. Each semi-transparent line represents one of the 45 tanks. The length of the experiment differed among temperature groups because sea-louse development rate increases with temperature. The fluctuations in the three lower tempeature groups (i.e., those that were manually adjusted rather than electronically) in the first ten days were primarily due to the influence of Hurricane Dorian on the ambient seawater temperature. Temperatures were recorded every three minutes by HOBO® Pendant® MX2201 loggers.


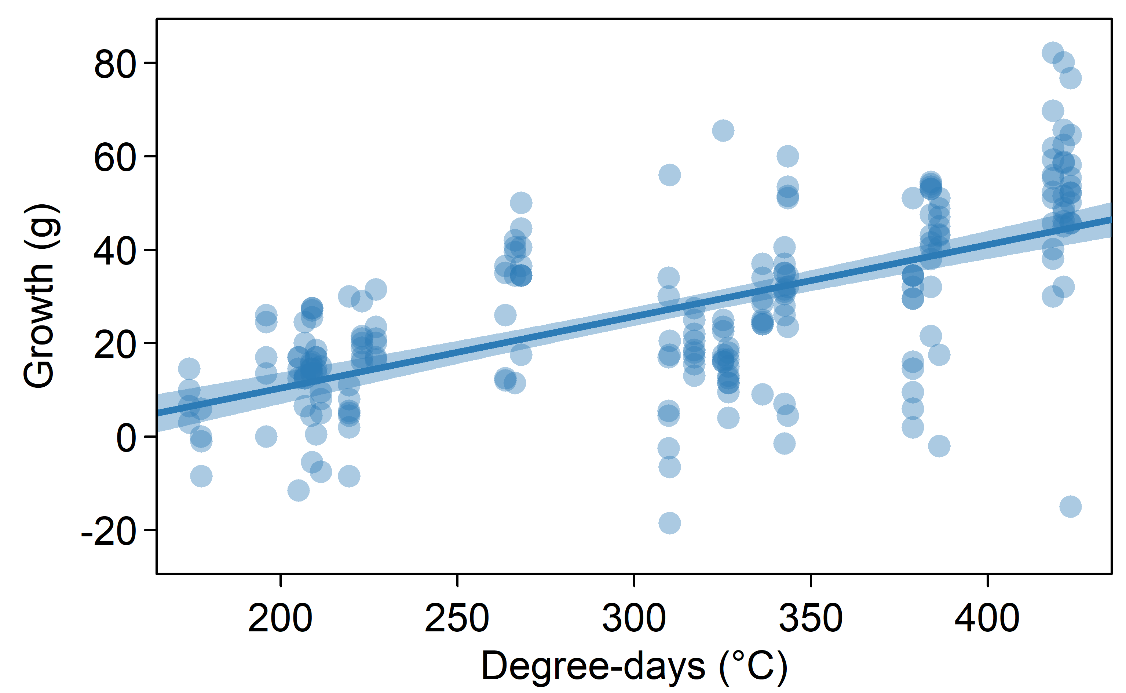


Fig. S6. Body growth during the experiment as of a function of degree-days for uninfested fish weighed during the midpoint and endpoint dissections. Initial size is not taken into account, contributing to extra variation in the amount of growth.


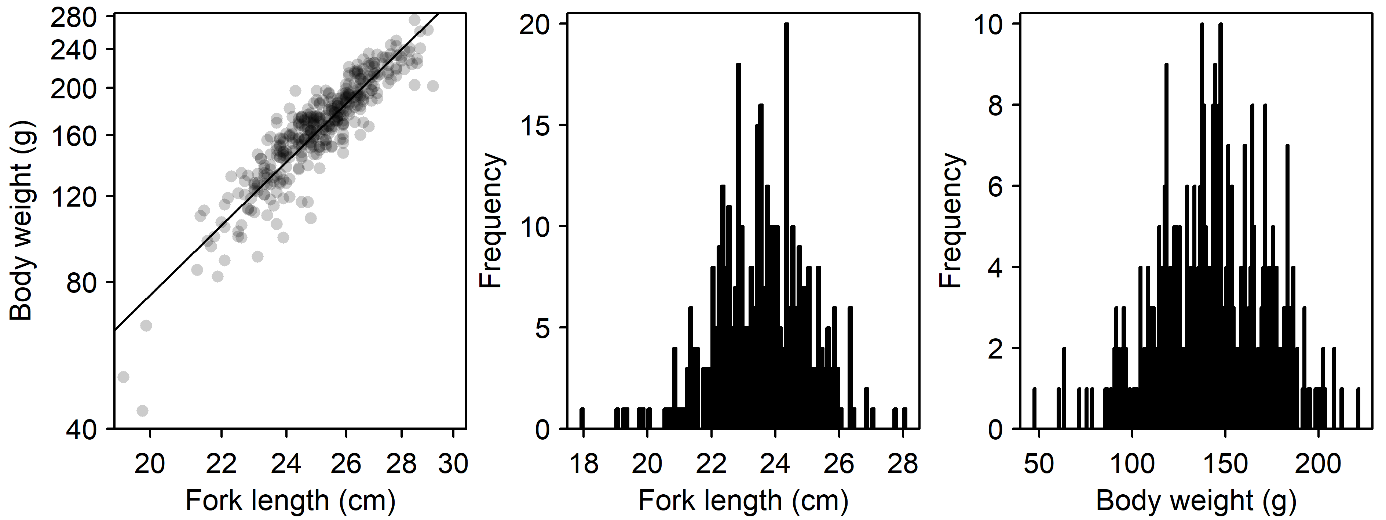


Fig. S7. Log-transformed weight-length relationship for the juvenile salmon at the start of the experiment (left panel), frequency histogram for the initial fork lengths (centre panel), and frequency histogram for the initial body weights (right panel).

Table S1. Summary statistics for the growth-rate models. Infestation (*inf*) had three levels (‘zero’, ‘low’, and ‘high’) and temperature (*temp*) was treated as a continuous covariate. Each model included a variance structure that allowed for unequal variance among infestation levels (by specifying a varIdent structure in the weights argument of the lme function in R) and a random effect on the intercept for tank number. Interaction terms (i.e., those with an ‘x’) included their main effects. ‘ΔAIC’ is the difference from the top model AIC and ‘Weight’ is the Akaike model weight. The results were robust with respect to the inclusion/exclusion of the individuals with the few individuals with likely measurement error.

| **Rank** | **Model** | **ΔAIC** | **Weight** |
| --- | --- | --- | --- |
| 1 | inf x temp^*^ | 0.00 | 0.99 |
| 2 | inf + temp | 9.64 | 0.01 |
| 3 | temp | 58.11 | 0.00 |
| 4 | inf | 91.14 | 0.00 |
| 5 | null | 100.17 | 0.00 |

^*^The marginal *R^2^* (i.e., the variance explained by the fixed effects alone) for the top model was 0.49 and the conditional *R^2^* (i.e., the variance explained by the entire model, including random effects) was 0.50 ^2^.

Table S2. Summary statistics for the change-in-condition models. Infestation (*inf*) had three levels (*zero*, *low*, and *high*) and temperature (*temp*) was treated as a continuous covariate. Each model included a random effect on the intercept for tank number. Interaction terms (i.e., those with an ‘x’) included their main effects and quadratic terms included their lower-order terms. ‘ΔAIC’ is the difference from the top model AIC and ‘Weight’ is the Akaike model weight. The results were robust with respect to the inclusion/exclusion of the individuals with the few individuals with likely measurement error.

| **Rank** | **Model** | **ΔAIC** | **Weight** |
| --- | --- | --- | --- |
| 1 | inf x temp^2^ | 0.00 | 0.83 |
| 2 | inf + temp^2^ | 3.13 | 0.17 |
| 3 | temp^2^ | 17.47 | 0.00 |
| 4 | inf x temp | 24.14 | 0.00 |
| 5 | inf + temp | 25.09 | 0.00 |
| 6 | temp | 33.48 | 0.00 |
| 7 | inf | 45.67 | 0.00 |
| 8 | null | 49.86\ | 0.00 |

^*^ The marginal *R^2^* (i.e., the variance explained by the fixed effects alone) for the top model was 0.25 and the conditional *R^2^* (i.e., the variance explained by the entire model, including random effects) was 0.29 [1].

Table S3. Summary statistics for the survival models. Infestation (*inf*) had three levels (*zero*, *low*, and *high*) and temperature (*temp*) was treated as a continuous covariate. Each model included a random effect on the intercept for tank number. Interaction terms (i.e., those with an ‘x’) included their main effects. ‘ΔAIC’ is the difference from the top model AIC and ‘Weight’ is the Akaike model weight.

| **Rank** | **Model** | **ΔAIC** | **Weight** |
| --- | --- | --- | --- |
| 1 | inf x temp^*^ | 0 | 0.86 |
| 2 | inf + temp | 3.69 | 0.14 |
| 3 | temp | 12.09 | 0 |
| 4 | inf | 26.72 | 0 |
| 5 | null | 32.93 | 0 |

^*^ We cannot report *R^2^* values for the top model because, unlike for generalized linear mixed-effects models (Table S2 and S3), the statistical framework for calculating *R^2^* values from mixed-effects Cox models has yet to be developed.

Table S4. Number of mortalities for each combination of infestation level and temperature; there were three tanks per combination. The number of fish at the start of the eperiment varied due to the mortalities pre-infestation.

| **Infestation level** | **Temperature group (°C)** | **Number of mortalities** | **Number of fish at start of experiment** |
| --- | --- | --- | --- |
| zero | 10 | 2 | 61 |
| zero | 13 | 1 | 58 |
| zero | 16 | 1 | 54 |
| zero | 19 | 2 | 54 |
| zero | 22 | 3 | 35 |
| low | 10 | 1 | 55 |
| low | 13 | 2 | 56 |
| low | 16 | 4 | 60 |
| low | 19 | 14 | 51 |
| low | 22 | 8 | 58 |
| high | 10 | 3 | 56 |
| high | 13 | 8 | 58 |
| high | 16 | 7 | 60 |
| high | 19 | 12 | 57 |
| high | 22 | 14 | 28 |

Table S5. Parameter estimates for the top growth-rate model. Infestation (*inf*) had three levels (*zero*, *low*, and *high*) and temperature (*temp*) was treated as a continuous covariate.

| Parameter name | Mean estimate ± standard deviation |
| --- | --- |
| intercept | 0.1182 ± 0.0164 |
| *inf_low_* | 0.0473 ± 0.0228 |
| *inf_high_* | 0.0069 ± 0.0221 |
| *temp* | -0.0065 ± 0.0010 |
| *inf_low_* : *temp* | -0.0050 ± 0.0014 |
| *inf_high_* : *temp* | -0.0039 ± 0.0014 |

Table S6. Parameter estimates for the top change-in-condition model. Infestation (*inf*) had three levels (*zero*, *low*, and *high*) and temperature (*temp*) was treated as a continuous covariate.

| Parameter name | Mean estimate ± standard deviation |
| --- | --- |
| intercept | 0.7431 ± 0.1155 |
| *inf_low_* | -0.0377 ± 0.1669 |
| *inf_high_* | -0.1931 ± 0.1701 |
| *temp* | 0.0328 ± 0.0151 |
| *temp^2^* | -0.0011 ± 0.0005 |
| *inf_low_* : *temp* | -0.0057 ± 0.0218 |
| *inf_high_* : *temp* | -0.0275 ± 0.0222 |
| *inf_low_* : *temp^2^* | -0.0002 ± 0.0007 |
| *inf_high_* : *temp^2^* | -0.0011 ± 0.0007 |

Table S7. Parameter estimates for the top survival model. Infestation (*inf*) had three levels (*zero*, *low*, and *high*) and temperature (*temp*) was treated as a continuous covariate.

| Parameter name | Exponentiated mean estimate ± standard deviation |
| --- | --- |
| *inf_low_* | 0.9494 ± 1.9998 |
| *inf_high_* | 2.2770 ± 1.8737 |
| *temp* | 1.2193 ± 0.0940 |
| *inf_low_* : *temp* | 1.0697 ± 0.1132 |
| *inf_high_* : *temp* | 1.0452 ± 0.1064 |

Table S8. Summary statistics for the alternate growth-rate models. These models were identical to the growth-rate models reported in the main text (Table S1), with the exception that the temperature correlate was the average temperature in each tank rather than the average temperature in each temperature treatment.

| **Rank** | **Model** | **ΔAIC** | **Weight** |
| --- | --- | --- | --- |
| 1 | inf x temp^*^ | 0.00 | 0.99 |
| 2 | inf + temp | 8.93 | 0.01 |
| 3 | temp | 56.02 | 0.00 |
| 4 | inf | 89.35 | 0.00 |
| 5 | null | 98.39 | 0.00 |

Table S9. Summary statistics for the alternate change-in-condition models. These models were identical to the growth-rate models reported in the main text (Table S2), with the exception that the temperature correlate was the average temperature in each tank rather than the average temperature in each temperature treatment.

| **Rank** | **Model** | **ΔAIC** | **Weight** |
| --- | --- | --- | --- |
| 1 | inf x temp^2^ | 0.00 | 0.81 |
| 2 | inf + temp^2^ | 2.96 | 0.19 |
| 3 | temp^2^ | 16.03 | 0.00 |
| 4 | inf x temp | 23.04 | 0.00 |
| 5 | inf + temp | 23.99 | 0.00 |
| 6 | temp | 31.62 | 0.00 |
| 7 | inf | 43.22 | 0.00 |
| 8 | null | 47.29 | 0.00 |

Table S10. Summary statistics for the alternate survival models. These models were identical to the growth-rate models reported in the main text (Table S3), with the exception that the temperature correlate was the average temperature in each tank rather than the average temperature in each temperature treatment.

| **Rank** | **Model** | **ΔAIC** | **Weight** |
| --- | --- | --- | --- |
| 1 | inf x temp^*^ | 0 | 0.87 |
| 2 | inf + temp | 3.76 | 0.13 |
| 3 | temp | 11.59 | 0 |
| 4 | inf | 25.74 | 0 |
| 5 | null | 31.94 | 0 |

**References**

1 Johnson, S., Blaylock, R., Elphick, J. & Hyatt, K. Disease induced by the sea louse (*Lepeophteirus salmonis*)(Copepoda: Caligidae) in wild sockeye salmon (*Oncorhynchus nerka*) stocks of Alberni Inlet, British Columbia. *Can. J. Fish. Aquat. Sci.* **53**, 2888-2897, (1996).

2 Nakagawa, S., Johnson, P. C. & Schielzeth, H. The coefficient of determination *R^2^* and intra-class correlation coefficient from generalized linear mixed-effects models revisited and expanded. *Journal of the Royal Society Interface* **14**, 20170213, (2017).
